# Improved genome-scale multi-target virtual screening via a novel collaborative filtering approach to cold-start problem

**DOI:** 10.1038/srep38860

**Published:** 2016-12-13

**Authors:** Hansaim Lim, Paul Gray, Lei Xie, Aleksandar Poleksic

**Affiliations:** 1 Department of Computer Science, Hunter College, The City University of New York, New York, New York 10065, United States; 2 Department of Computer Science, University of Northern Iowa, Cedar Falls, Iowa 50614, United States; 3 Ph.D. Program in Computer Science, Biochemistry and Biology, The Graduate Center, The City University of New York, New York, New York 10065, United States

## Abstract

Conventional one-drug-one-gene approach has been of limited success in modern drug discovery. Polypharmacology, which focuses on searching for multi-targeted drugs to perturb disease-causing networks instead of designing selective ligands to target individual proteins, has emerged as a new drug discovery paradigm. Although many methods for single-target virtual screening have been developed to improve the efficiency of drug discovery, few of these algorithms are designed for polypharmacology. Here, we present a novel theoretical framework and a corresponding algorithm for genome-scale multi-target virtual screening based on the one-class collaborative filtering technique. Our method overcomes the sparseness of the protein-chemical interaction data by means of interaction matrix weighting and dual regularization from both chemicals and proteins. While the statistical foundation behind our method is general enough to encompass genome-wide drug off-target prediction, the program is specifically tailored to find protein targets for new chemicals with little to no available interaction data. We extensively evaluate our method using a number of the most widely accepted gene-specific and cross-gene family benchmarks and demonstrate that our method outperforms other state-of-the-art algorithms for predicting the interaction of new chemicals with multiple proteins. Thus, the proposed algorithm may provide a powerful tool for multi-target drug design.

Drug action is a complex process. A drug starts to take effect on a biological system when it interacts with its targets. However, a drug rarely binds to a single target. Multiple target binding, i.e., polypharmacology, is a common phenomenon^1^. To understand how polypharmacology leads to the alteration of the cellular state through gene regulation, signaling transduction, and metabolism, and ultimately causes the change of the physiological or pathological state of the individual, a multi-scale modeling approach is needed^2^,^3^. In the framework of multi-scale modeling, drug targets are first predicted on a genome scale. Then these drug targets along with the non-targeted genes associated with a particular phenotype are mapped to a biological network to model, simulate, and predict the phenotypic response of drug action^4^,^5^,^6^,^7^,^8^,^9^. Thus, the accurate and efficient prediction of genome-scale drug-target interactions is critical to reveal the genetic, molecular, and cellular mechanisms of drug action.

To date, few computational tools that support the discovery and application of multi-target therapies are available. The existing computational methods are tailored for single-target drug design and can be classified into two groups. The first group consists of methods that exploit structural information of a protein binding site, trying to synthesize a suitable compound de novo^10^,^11^. The methods from the second group search large databases of candidate compounds through a process known as virtual screening^12^,^13^. Guiding criteria for virtual screening include complementary geometries as well as favorable physical and chemical properties of the candidate compounds and the proteins’ binding sites^14^. Although theoretically appealing, both approaches face significant obstacles, which include:

- Computational complexity, due to the number of possible ligand conformations (for de novo methods) and the enormous size of compound libraries (for virtual screening),
- Inability to adequately normalize the objective function in order to properly rank numerous solutions (i.e., ligands constructed de novo for the methods in the first group or ligands extracted from the compound libraries for the methods from the second group).

Recent years have seen the development of knowledge-based methods for protein-ligand interactions^15^,^16^,^17^. These algorithms rely on statistical and mathematical procedures to build upon the existing knowledge stored in the databases of known interactions^18^. In attempt to come up with more efficient and more accurate algorithms, biomedical researchers are starting to incorporate a variety of techniques from many different and seemingly unrelated fields. Recommender systems, which are used in the movie industry to predict users’ preferences for movies, are finding their ways into computational molecular biology and biomedical research. In particular, techniques such as collaborative filtering^19^, compressed sensing^20^, and low-rank matrix completion^21^ have been successfully applied to discover novel protein-protein interactions^22^ and to reconstruct gene regulatory networks^23^. However, most of these methods have only sub-optimal performance in predicting preferences of new items. A computational method able to find targets for compounds with no available interaction data would help overcome the inaccuracy and complexity of de novo ligand design and virtual screening.

In this paper we present COSINE (COldStartINtEractions) - a statistical framework and a corresponding computational method for multi-target virtual screening via the “one-class collaborative filtering” technique. Our program exploits existing knowledge and databases of known interactions as well as the sequence similarities between proteins and structural similarities between drug molecules to suggest potential targets for new chemicals. Among unique aspects of our work are position specific weights, impute values, and a novel weighted-profile procedure for improving target prediction for novel chemicals. The accuracy of COSINE is validated in blind benchmarks that utilize well-known and publicly available resources. Our data shows that COSINE clearly outperforms numerous state-of-the-art methods for the same problem in several different tests and with respect to different accuracy measures. The algorithm is freely available at http://bioinfo.cs.uni.edu/COSINE.html.

## Methods

In a typical recommender system, user rating is expressed using different scores (e.g. 1–5 scale used by Netflix’s users to rate movies). However, the nature of available data for protein-chemical interactions is different. Often times, only “positive” data consisting of known and validated interactions is available but there is no straightforward way of distinguishing “negative” scores (no interactions) from the missing data. The underlying binary score system (1 for interacting pairs and 0 otherwise) necessitates a deviation of the computational models used for protein-chemical interactions from the classical recommender models. COSINE belongs to the category of one-class collaborative filtering methods^24^,^25^ since it does not treat all missing data as negative data. The protein-chemical interactions are predicted using the “low-rank matrix factorization” technique. More formally, given an incomplete matrix *R* of observed interactions, with *m* rows, representing targets, and *n* columns, representing chemicals, our algorithm decomposes *R* into a product of two lower dimension matrices of dimensions *m* × *r* and *r* × *n, r* ≪ min(*m, n*). The component matrices correspond to proteins’ and chemicals’ latent preferences. The assumption is that the set of proteins (respectively, chemicals) under consideration can be divided into a relatively small number of subsets with different proteins from the same subset exhibiting the same preferences to chemicals. Our algorithm takes account of the fact that related proteins, such as those with similar amino-acid sequences or similar three dimensional structures, exhibit similar preferences to chemicals and vice versa (structurally similar chemicals show similar preferences to proteins).

### Statistical framework

COSINE is a dual-regularized, one-class collaborative filtering method^25^ that can employ either logistic or linear factorization. Our method can be thought of as a multi-directional extension of some recently described matrix factorization techniques for making recommendations^26^,^27^. Specifically, let *m* and *n* represent the number of proteins and chemicals, respectively, and let *R* = (*r*_*i*,*j*_) be a *m* × *n* matrix of protein-chemical interactions


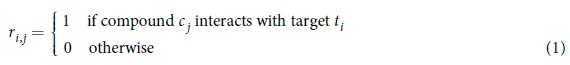


In protein-chemical interaction studies, the binary matrix *R* is typically incomplete. While each nonzero entry *r*_*i*,*j*_ = 1 signifies a known interaction, the meaning of each zero entry *r*_*i*,*j*_ = 0 is ambiguous in that there can be either no interaction between the target *t*_*i*_ and the compound *c*_*j*_, or, alternatively, that an interaction exists but it has never been verified experimentally. Thus, the goal is to predict the missing entries (i.e., to reclassify all unknown entries of the matrix *R*).

Building upon the general low-rank matrix factorization framework, COSINE approximates the probability of each chemical interacting with each target by mapping both chemicals and proteins to a common latent space of reduced dimensionality. The assumption here is that the number of factors influencing protein-chemical interactions is relatively small or, more formally, that the matrix of protein-chemical interactions is of low rank and, therefore, that it can be written as the product *FG*^*T*^ of two matrices *F* and *G* of dimensions *m* × *r* and *n* × *r*, respectively, where *r* ≪ min (*m, n*) represents the number of latent factors. While our program is capable of performing either linear or logistic factorization, in the rest of this paper we will focus on logistic factorization, because it allows for an elegant statistical treatment.

Following Steck^28^, we first consider the loss function:


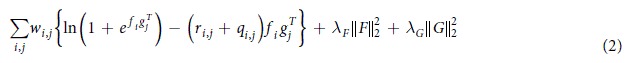


where *f*_*i*_ and *g*_*j*_ denote the *i*^*th*^ and *j*^*th*^ row (latent vector) of the matrices *F* and *G*, respectively, *w*_*i*,*j*_ are the position specific weights on interaction scores, *q*_*i*,*j*_ are the so-called “imputation values”^25^, *λ*_*F*_, *λ*_*G*_ are tunable parameters and 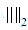 denotes the Frobenius norm. The regularization terms 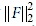 and 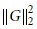 are included to prevent over-fitting.

COSINE extends several other methods for the protein-chemical interaction prediction^16^,^27^, in at least two directions. Namely, the algorithm allows not only for the imputation of interaction values but also for different weighting of the interaction entries. In fact, to the best of our knowledge, COSINE is the only method for protein-chemical interaction prediction that employs position-specific weight and imputation values.

To provide insight into the motivation behind our method, consider, for instance, an ambiguous case where *r*_*i*,*j*_ = 0 but some new experimental evidence suggests that the chemical *c*_*j*_ might interact with protein *t*_*i*_. We can utilize this new knowledge by setting *q*_*i*,*j*_ = 1 while lowering the corresponding weight *w*_*i*,*j*_ to account for any uncertainty in the imputed value. A more thorough justification of the objective function (2) is given below. For a less general case, we refer the reader to Johnson^26^ and Liu *et al*.^27^.

### Position specific weights and impute values

To derive the function (2) analytically, let *e*_*i*,*j*_ be the event that the compound *c*_*j*_ interacts with the target *t*_*i*_. Assume that the probability distribution of *e*_*i*,*j*_ is logistic. In other words, assume that the probability *p*_*i*,*j*_ assigned to *e*_*i*,*j*_ is given by


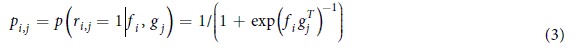


Recall also that *w*_*i*,*j*_ reflects the confidence in the entry *r*_*i*,*j*_ of the interaction matrix *R*. More precisely, higher weights are assigned to protein-chemical pairs (*t*_*i*_, *c*_*j*_) which are known to interact (*r*_*i*,*j*_ = 1), while lower values of *w*_*i*,*j*_ are given to pairs for which *r*_*i*,*j*_ = 0. To put it differently, a high number of positive training examples corresponds to each interacting pair while a lower number of negative training examples corresponds to each non-interacting (or unknown) pair. Hence, the likelihood of *r*_*i*,*j*_ + *q*_*i*,*j*_ given *f*_*i*_ and *g*_*j*_ can be written as


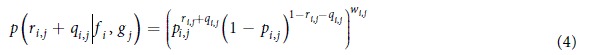


or, at the matrix level,


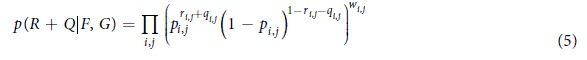


As in Steck^28^, the probability *p(F, G*|*R* + *Q*) can be derived through the Bayesian inference


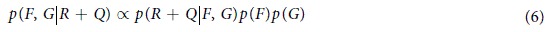


Finally, we derive the loss function (2) by taking the negative logarithm of (6), while assuming the Gaussian distribution of the entries of *F* and *G*^26^. Thus, in contrast to linear loss function^25^, namely


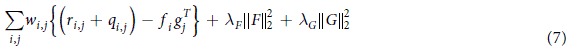


the logistic loss function (used by default in our method) has an explicit probabilistic interpretation.

### Dual regularization from proteins and chemicals

To increase the accuracy of protein-chemical interaction prediction, we further extend the loss function (2) to account for the fact that similar chemicals are likely to interact with similar targets. Formally, let *M* = (*m*_*i*,*j*_) be the matrix of pair-wise target similarity scores, where each entry *m*_*i*,*j*_ represents the similarity between the proteins *t*_*i*_ and *t*_*j*_, and let *N* = (*n*_*i*,*j*_) be the matrix of pair-wise compound similarity scores. The affinity of similar chemicals to bind similar proteins is accounted for by minimizing the protein homophily


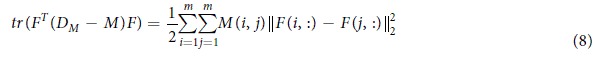


and the compound homophily


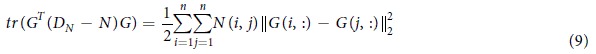


Incorporating the regularization terms (8) and (9) above into (2), and introducing two additional tunable parameters, *λ*_*M*_ and *λ*_*N*_, our loss function becomes


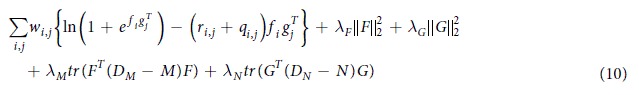


**Figure 1:**
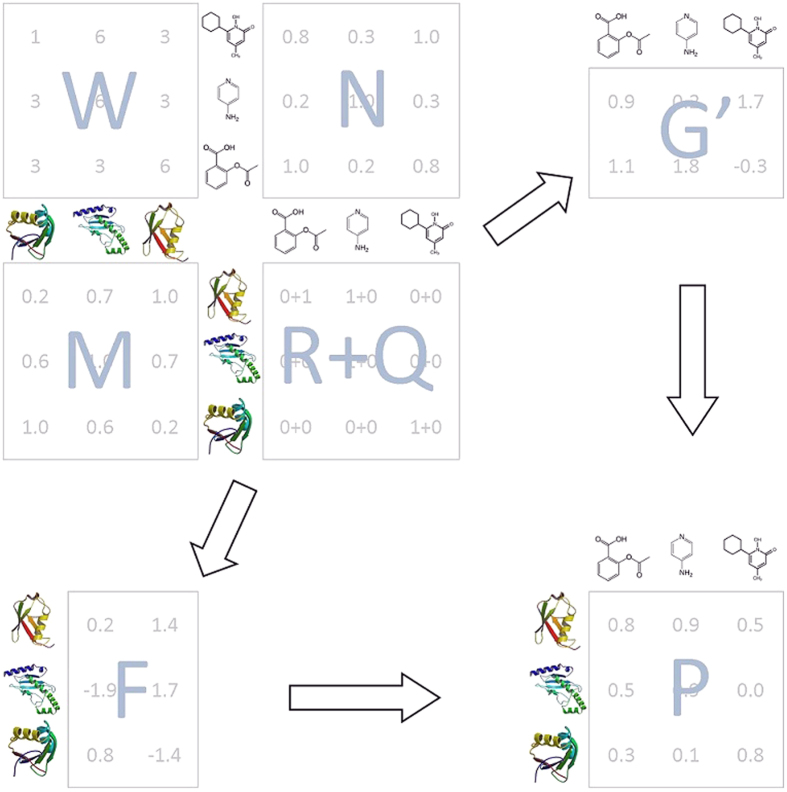
The components of the loss function. INPUT: the sum of the interaction and impute matrices R + Q; the weight matrix W; the protein similarity matrix M; the chemical similarity matrix N. OUTPUT: the matrix of protein latent preferences F; the matrix of chemical latent preferences G; the matrix of predicted interaction probabilities 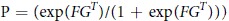.

Figure 1 provides a toy example illustrating various components of the loss function.

In practice, the entries *m*_*i*,*j*_ of the matrix *M* typically represent the sequence similarity of the primary structures of proteins *t*_*i*_ and *t*_*j*_, as measured, for example, by the normalized Smith- Waterman alignment score or by the PSI-BLAST e-value^29^. Alternatively, the values *m*_*i*,*j*_ can be chosen to represent the three-dimensional similarity of the proteins’ tertiary structures. Similarly, each *n*_*i*,*j*_ represents the similarity score for the compounds *c*_*i*_ and *c*_*j*_, as measured, for instance, by the Tanimoto score^30^ or by the similarity of *c*_*i*_′*s* and *c*_*j*_′*s* pharmacological profiles^15^.

Note that the partial derivatives of (10) can be written as


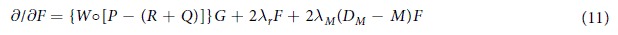



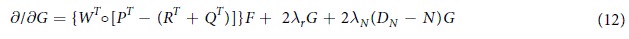


where 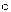 represents the Hadamard product.

There are several ways to minimize the loss function (10)^25^,^26^,^27^. Similar to Liu *et al*.^27^, COSINE uses the AdaGrad - an iterative gradient descent method^31^.

### Weighted-profile approach for virtual screening

The most challenging task in protein-chemical interaction prediction is known as the “cold-start problem”. The goal is to predict interactions of chemicals (or targets) for which no interaction data is available. COSINE implements a modified version of the “weighted profile” method^32^,^33^ in which the latent preferences for a new protein (the rows of *F*) are computed as the sum of the latent preferences for that protein (calculated by the iterative minimization procedure, described above) and the latent preferences of *J* most similar proteins (those with available interaction data). More specifically, we set the *i*^*th*^ row of the matrix *F* for the new target *t*_*i*_ to


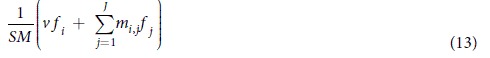


where *f*_*j*_ is the *j*^*th*^ row of *F* (representing the latent preferences of the target *t*_*j*_), *v* is the weight parameter and *m*_*i*,*j*_ is the similarity score of the targets *t*_*i*_ and *t*_*j*_. The normalization factor *SM* is set to 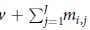.

The latent preferences for new chemicals (rows of *G*) are computed in the same way, using the compound similarity scores *n*_*i*,*j*_, namely.


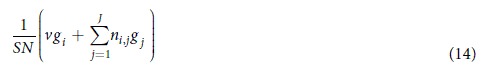


where 
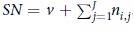
.

### Algorithmic details

COSINE minimizes the loss function (10) twice. The first time around, all imputation values *q*_*i*,*j*_ are set to zero. The initial weights are set to 6 if *r*_*i*,*j*_ = 1 and 1 otherwise, to reflect our increased confidence in experimentally verified interactions and lesser confidence in values *r*_*i*,*j*_ = 0 (absent or unknown interaction). In the second iteration of the algorithm, the weight (which might be interpreted as the confidence in the value) of *r*_*i*,*j*_ is increased by one if the computed probability of interaction *p*_*i*,*j*_ is either too small or too large (more details are given in the Supplementary Table S1). The imputation values *q*_*i*,*j*_ are adjusted in such a way that each entry of the new input matrix of interactions (namely *r*_*i*,*j*_ + *q*_*i*,*j*_) is set to 1 if the probability of the interaction *p*_*i*,*j*_ computed in the first step is high (Supplementary Table S1). Otherwise, it is set to max(*r*_*i*,*j*_, *p*_*i*,*j*_). We take max(*r*_*i*,*j*_, *p*_*i*,*j*_) rather than *p*_*i*,*j*_ since our underlying assumption is that the true interactions have been experimentally verified and hence the nonzero values of *r*_*i*,*j*_ should be taken account of in the second step.

## Results

To validate the algorithm, we compared it to a number of different methods for the same problem, namely KBMF2K^34^, WNN^33^, WNN-GIP^33^, NetLapRLS^35^, BLM-NII^36^, CMF^37^, NRLMF^27^, PRW^38^, REMAP^39^, Chem08^32^, Pharm10^15^, DASPfind^40^, NRWRH^41^ and HGBI^42^ in several different benchmarks, namely Yam^32^, Yam^15^, and ZINC^39^.

### Benchmark #1

We first tested the accuracy of our algorithm in the classic Yam08 benchmark designed by Yamanishi *et al*.^32^. In this benchmarking experiment, which uses two different accuracy measures (AUPR and AUC), each dataset consists of four classes of targets: Enzymes, Ion Channels, GPCR’s and Nuclear Receptors (Supplementary Table S2).

In order to compare COSINE directly to the methods previously tested in this benchmark (KBMF2K, WNN and WNN-GIP) we performed a 5-fold cross-validation on the set of chemicals. More specifically, for each protein class, the set of chemicals was split into 5 subsets of approximately equal size and each subset was taken in turn as a test set. As described in van Laarhoven and Marchiori^33^, the training was performed on the remaining 4 subsets. The summary of the methods’ accuracies, as measured by the area under the Precision-Recall curve (AUPR) and the area under the ROC curve (AUC), is given in Table 1. As seen in this table, while WNN method compares favorably to COSINE in the Enzyme class test, COSINE outperforms all of its competitors in all other target classes, most of the time, significantly. Moreover, the average AUPR and AUC scores achieved by COSINE exceed the average accuracies achieved by any other method tested in this benchmark.

### Benchmark #2

Some methods for protein-chemical interaction prediction have been tested in Yam08 benchmark that uses 10-fold instead of 5-fold cross validation. To compare COSINE with those algorithms we modified the testing procedure and (similar to Liu *et al*.^27^) ran 5 rounds of 10-fold cross-validation on the set of chemicals. Our findings are summarized in Table 2. As seen in these tables, COSINES achieves the best overall result, as measured by AUPR and AUC metrics. In contrast to 5-fold cross validation experiment, our method outperforms WNN-GIP in the Enzyme class benchmark with respect to both measures, but achieves a slightly lower AUPR than NRLMF (0.346 vs. 0.358).

It is interesting to note that COSINE’s closest competitor in this test, namely NRLMF, also employs logistic factorization. However, unlike COSINE, the NRLMF method sets the weights globally, uses no imputation values and employs a different weighted profile scheme for cold start predictions. A different comparison of the two methods, using a different test sets and a different accuracy measure is presented in the subsection Benchmark #5 below.

### Benchmark #3

Our next benchmarking data set, Yam^15^, has been constructed from the previous one^32^ by extracting only the data corresponding to the compounds with available pharmacological profiles (Supplementary Table S3). Consequently, this benchmark mandates that all methods submitted use the similarity scores between pharmacological profiles computed by Yamanishi *et al*.^15^, in place of Tanimoto scores.

Strictly speaking, the only two algorithms that have been tested previously in the Yam10 benchmark using cross-validation on chemicals are the Yamanishi’s 2008 algorithm, and its improved version, based on similarity of compounds’ pharmacological profiles. Cobanoglu *et al*. have submitted their probabilistic matrix factorization method to a similar test^16^, but their analysis was performed under conditions conceptually different from cross-validation on chemicals. For this reason, we do not include the results of Cobanoglu *et al*. here. The results of KBMF2K^34^ are not suitable for the direct comparison with COSINE either, since they are obtained on the Yam08 benchmark and not on Yam10. As shown in the Supplementary Table S4, COSINE compares favorably to the other two methods tested, irrespective of the drug similarity matrix used (Tanimoto similarity or similarity of drugs’ pharmacological profiles).

### Benchmark #4

We have also compared COSINE to methods previously tested in the leave-one-out cross validation experiment that uses the Top 1 predictions as the accuracy criterion. Following the protocol described in the DASPfind paper^40^, for each target set and each drug under consideration, we removed all of the drug’s known interactions and tried to retrieve them as Top1 predictions. As shown in Table 3, COSINE retrieves more interactions as Top1 predictions than any other method submitted to this benchmark. Although we have not trained the parameters of COSINE for this benchmark (we used the default ones found to work the best in the previous tests) it is reasonable to believe that the superior performance of COSINE over the other three methods is due to the fact that our algorithm has been explicitly developed to predict targets for new drugs (cold start). In contrast, the other three methods are tailored to not only “cold start” but also to “off-target” predictions.

### Benchmark #5

Lastly, we compared the performance of COSINE to selected methods in the extensive ZINC benchmark. To generate the ZINC test sets, the ZINC data^43^ was filtered by IC50 ≤ 10 μM. This process yielded 31735 unique chemical-protein associations for 3,500 proteins and 12,384 chemicals. Cell-based tests and proteins appearing in protein complexes were excluded as well as proteins with unavailable primary sequences. Protein sequences were taken from UniProt^44^. Protein-protein similarity scores were calculated using BLAST.

The ability of different algorithms to “rediscover” interactions was measured by “hiding” (setting to zero) the corresponding entries in the protein-chemical interaction matrix. To perform “cold start” analysis on ZINC data, we identified a set of chemicals having only one known target. The resulting set was further divided based on two criteria: 1) the number of chemicals the target proteins are associated with, and 2) the maximum chemical-chemical similarity score for the chemical in the dataset, with 0.1 increments. Each set was further subdivided into two subsets of approximately equal size, the test set (Supplementary Table S5) and the training set.

**Figure 2:**
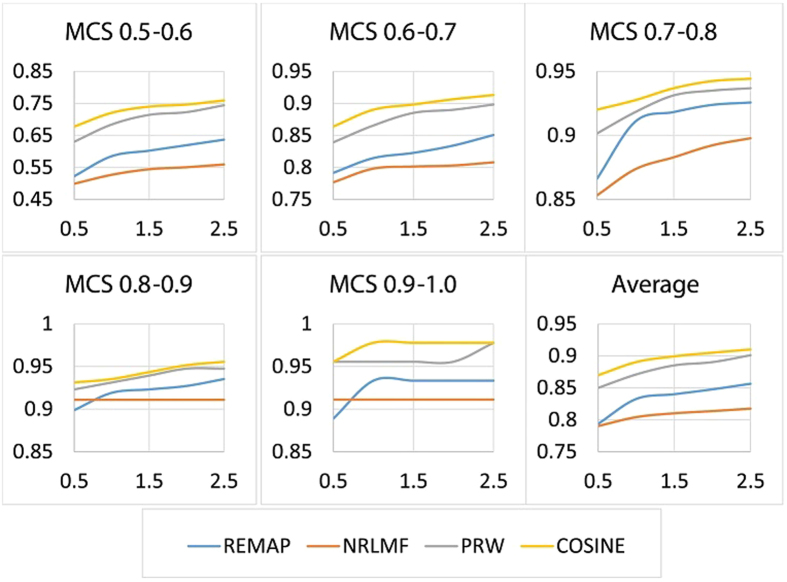
ZINC benchmark MCS. The True Positive Rate (TPR) at top *r*% predictions 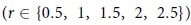 with varying number of (maximal) chemical structural similarity (MCS).

To provide for a conceptually different test, the ZINC benchmark uses the True Positive Rate (Recall or Recovery) at the top *r*% 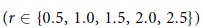 of predictions for each chemical as the benchmarking measure. The Recall (Recovery) is defined as *Recall* = *TP*/*CP*, where *TP* and *CP* represent the total number of true and condition positives, respectively. Since there is a total of 3,500 targets, the *r*% of predictions include (35 · *r*)^th^ or higher ranked target for each chemical. For instance, the True Positive Rate (TPR) of 0.7 at the 35^th^ cutoff rank (top 1%) means that 70% of the total tested positive pairs were ranked 35^th^ or better for the tested chemicals. Using TPR in place of AUC allows us to assess the performance of COSINE from a different angle. In particular, it is informative to compare COSINE head-to-head to NRLMF again, since, according to the results by Liu *et al*.^27^, the accuracies of NRLFM are significantly higher than KBMF2K, CMF, and WNN-GIP. Aside from NRLMF, we also analyzed the accuracy of one of the most popular and most widely used method for the cold start problem, based on the Parzen–Rosenblatt window (PRW) approach^38^. PRW is a highly accurate chemical structure-based target prediction method that uses neither the information obtained from proteins nor from the interactome. Finally, we submitted to the ZINC test a version of the COSINE algorithm, called REMAP, which uses linear (instead of logistic) factorization and global (instead of position specific) weights. Comparison with the latter algorithm (which has been used by our group for drug “off-target” prediction) is particularly useful since it illustrates the contribution of novel features of COSINE to its overall accuracy. Figures 2 and3 demonstrate the performance of different algorithms in the ZINC test, as measured by Recall at the top *r*% of predictions for various values of *r*. The significant performance advantage of COSINE over REMAP illustrates the benefits of using local weights, logistic (as opposed to linear) factorization and a weighted profile approach for novel drugs and novel targets.

### Additional analyses

**Figure 4:**
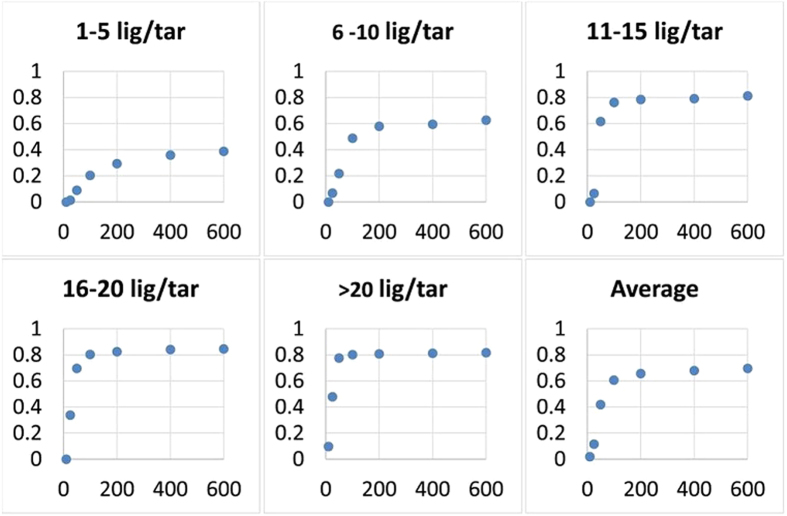
Accuracy over iterations. The accuracy of COSINE (TPR at top 1%; y-axis) as a function of the number of iterations (x-axis) in different subsets of the ZINC benchmark (1–5, 6–10, 11–15, 26–20, and >20 ligands per target).

We studied how the number of iterations in the matrix factorization step influences the accuracy of our algorithm. In our experiments, the convergence is attained after about 50 iterations for smaller data-sets (such as *Nuclear Receptors* test set) while a larger number of iterations (100–600) is needed to achieve comparable accuracy on larger data sets (such as *Enzymes* or *ZINC* test). As seen in Fig. 4, for very large data sets, such as ZINC, the added value has a low diminishing return after ~500 iterations. Thus, we opted for a reasonable speed-accuracy tradeoff of 600 iterations. Increasing the number of iterations further renders the algorithm computationally infeasible. A proper adjustment for the number of iterations results in the runtime comparable to other methods (Supplementary Table S6).

**Figure 5:**
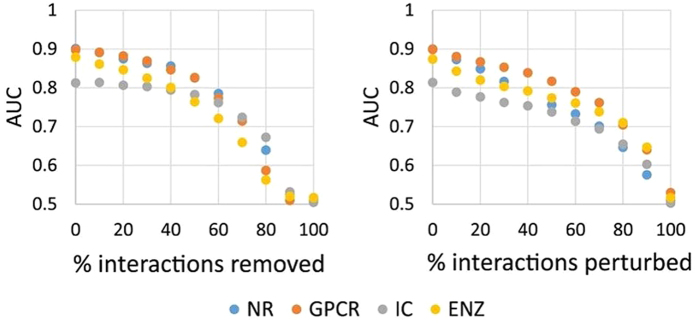
Accuracy as a function of noise. The accuracy of COSINE in the 10-fold CV Yam08 benchmark as a function of the amount of missing interaction data (left) and as a function of the amount of incorrect interaction data (right).

We also studied how COSINE performs in less than ideal settings, for instance, as a function of noise due to invalid or insufficient interaction data. We recorded the AUC values obtained on four target classes (NR, GPCR, Ion Channels and Enzymes) as a function of missing interaction data and as a function of incorrect interaction data. As shown in Fig. 5, our method is able to compensate a significant fraction of incorrect or missing data, due to the “low-rank matrix completion” technique built into the algorithm. This technique assumes that drugs’ preferences to targets are determined by a relatively small number of interaction patterns. Explicitly imposing the rank constraint in the loss function (as done in COSINE and some other matrix factorization methods) results in eliminating erroneous interactions, those that cannot be explained by the small dimensionality of the space of latent preferences.

## Discussion and Conclusion

Historically rational drug design has been characterized through identifying a single disease associated target and discovering exquisitely selective drugs against that target. Unfortunately, this one-drug-one-gene approach has been of limited success. This failure is manifest in the current issues facing the drug industry with near empty pipelines and costly post-market withdrawals. New methodologies are called for. Polypharmacology, which focuses on searching for multi-targeted drugs to perturb disease-causing networks instead of designing selective ligands to target individual proteins, has emerged as a new drug discovery paradigm^45^.

Computational methods that can assist polypharmacology are of key importance in drug development. *In-silico* protein-chemical interaction prediction has proven useful in *drug-repurposing (drug-repositioning*), an area of drug discovery that aims to find new therapeutic indications for known, FDA approved drugs^46^,^47^. Drug repurposing and other rational and structure-based drug design approaches are getting increased attention in the pharmaceutical industry as the cost of bringing a new drug to the market is approaching $1 billion^48^. A significant portion of the drug development cost is attributed to the inability of many candidate drug compounds to pass stages II and III of clinical trials, which is due to their insufficient efficacy and/or increased toxicity. Hence, the drug discovery pipeline can be made more efficient by taking advantage of a systematic, rational approach. This strategy assumes an automated prediction and analysis of interactions on a large scale, carried out by comparing large subsets of the proteome against a wide array of existing and candidate drug compounds.

Selected statistical techniques, including recommender systems, known as “low-rank matrix completion” and “collaborative filtering”, have been successfully used to predict protein-protein interactions^49^ and to identify the gene clusters from the microarray data^50^. However, to date, the use of these systems in predicting protein-chemical interactions has been limited, due to their limitations in ability to accurately predict interactions of new compounds and new targets.

We introduce a computational method for predicting protein-chemical interactions based on matrix factorization. Our method builds upon “collaborative filtering” - a widely used statistical technique for making recommendations to utilize existing knowledge stored in the databases of known interactions. By incorporating the weighting and imputation of the interaction data, as well as the dual regularization from both chemicals and targets, COSINE is able to exceed accuracy of other state-of-the-art methods for the same problem.

Our algorithm integrates chemoinformatics (chemical structural similarity), bioinformatics (protein sequence similarity) and a drug-target network (in form of matrix completion) approaches. Utilizing chemical structural similarity has proven useful (and has been widely applied) in the drug discovery for single-target virtual screening. Incorporating protein sequence similarity has shown promises in the prediction of drug off-targets^51^,^52^,^53^,^54^. Our drug-target network approach, formulated as a matrix completion problem, has been successfully applied to recommender system, which improve the performance of off-target prediction, especially when the chemoinformatics method fails.

The publically available chemogenomics data, on which all of existing virtual screening methods are inherently based, is incomplete and noisy. The missing interaction data is predicted in COSINE by completing the input interaction matrix, while biased and noisy data is filtered out by selecting the objective function that minimizes the rank of the output matrix of predicted interactions.

We recognize that, even though the ROC and PR curves may give a global estimation of the false positive rate for a prediction in the certain rank given by existing virtual screening algorithms, they may be not adequate for a risk-sensitive drug discovery application. In addition, in bio- and chemo-informatics applications, non-nested CV model is known to bias the parameters to the data set. Thus, other approaches to assessing reliability for specific new cases (including the label permutation and/or nested CV approach) will be extremely useful. We have developed several methods, e.g. ENTS^55^ and case-based reasoning^56^,^57^ for this purpose. In our on-going work, we plan to integrate these methods into the COSINE algorithm.

## Additional Information

**How to cite this article**: Lim, H. *et al*. Improved genome-scale multi-target virtual screening via a novel collaborative filtering approach to cold-start problem. *Sci. Rep.* **6**, 38860; doi: 10.1038/srep38860 (2016).

**Publisher's note:** Springer Nature remains neutral with regard to jurisdictional claims in published maps and institutional affiliations.

## Supplementary Material

Supplementary Information

## Figures and Tables

**Figure 3:**
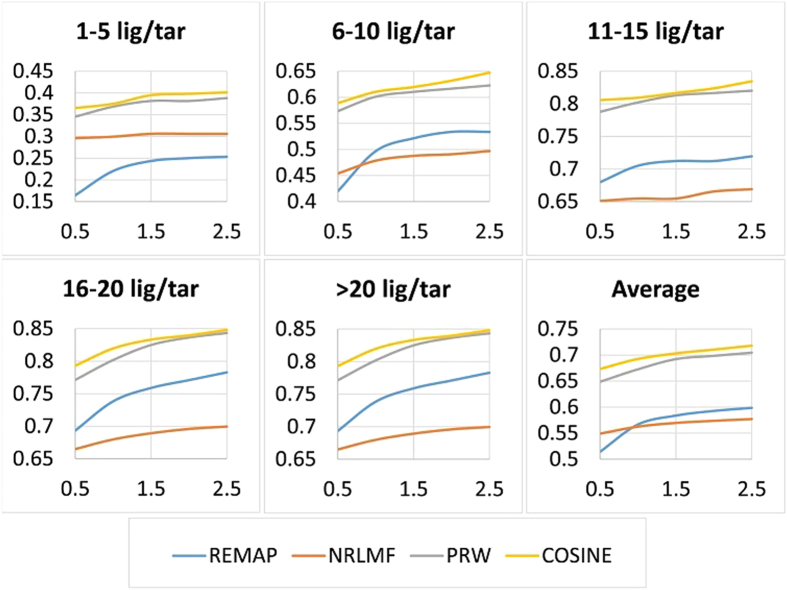
ZINC benchmark LT. The True Positive Rate (TPR) at top *r*% predictions 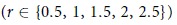 with varying number of ligands per target (LT).

**Table 1:** 5-fold cross-validation on Yam08 dataset.

	KBMF2K	WNN	WNN-GIP	COSINE
**AUPR**				
N. Recept.	*0.354*	*0.434*	0.456	0.511
GPCR	0.347	*0.308*	*0.311*	0.354
Ion Ch.	*0.245*	*0.249*	*0.233*	0.322
Enzyme	0.287	0.299	0.280	0.289
AVERAGE	**0.308**	**0.323**	**0.320**	**0.369**
**AUC**				
N. Recept.	*0.810*	*0.788*	*0.839*	0.901
GPCR	*0.840*	*0.848*	*0.872*	0.889
Ion Ch.	0.802	*0.757*	*0.775*	0.807
Enzyme	*0.812*	*0.819*	0.861	0.852
AVERAGE	**0.816**	**0.803**	**0.837**	**0.862**

The best results are underlined. Cases where COSINE significantly outperforms the competitor (t-test, p < 0.05) are shown in italic. The results for other methods were taken from van Laarhoven and Marchiori^33^.

**Table 2:** 10-fold cross-validation on Yam08 dataset.

	BLM-NII	CMF	KBMF2K	NetLapRLS	NRLMF	WNN-GIP	COSINE
**AUPR**							
N. Recept.	*0.438*	*0.488*	*0.477*	*0.417*	0.545	0.504	0.548
GPCR	*0.315*	*0.365*	*0.366*	*0.229*	*0.364*	*0.295*	0.397
Ion Ch.	*0.302*	*0.286*	*0.308*	*0.200*	0.344	*0.258*	0.359
Enzyme	*0.253*	*0.229*	*0.263*	*0.123*	0.358	0.278	0.346
AVERAGE	**0.327**	**0.342**	**0.354**	**0.242**	**0.403**	**0.334**	**0.410**
**AUC**							
N. Recept.	*0.799*	*0.818*	*0.844*	*0.789*	0.900	0.890	0.914
GPCR	*0.838*	*0.857*	*0.839*	*0.817*	0.895	*0.891*	0.902
Ion Ch.	*0.796*	*0.743*	*0.799*	*0.757*	0.813	0.797	0.826
Enzyme	*0.813*	*0.829*	*0.713*	*0.786*	0.871	0.882	0.888
AVERAGE	**0.812**	**0.812**	**0.799**	**0.787**	**0.870**	**0.865**	**0.883**

The best results are underlined. Cases where COSINE significantly outperforms the competitor (t-test, p < 0.05) are shown in italic.

The results for other methods were taken from Liu *et al*.^27^.

**Table 3:** The percentage of correct Top 1 predictions in Yam08 LOOCV benchmark.

	NRWRH	HGBI	DASPfind	COSINE
N. Recept.	31.48	46.3	51.85	55.56
GPCRs	25.56	42.15	51.12	53.36
Ion Chann.	33.33	35.71	44.28	54.29
Enzymes	18.65	43.6	49.66	56.4
AVERAGE	**27.26**	**41.94**	**49.23**	**54.9**

The best results (highest percentage of correct Top 1 predictions) are underlined.
