# Supplementary material for: Improved genome-scale multi-target virtual screening via a novel collaborative filtering approach to cold-start problem
Source: Sci Rep. 2016 Dec 13;6:38860. doi: 10.1038/srep38860 (PMC5153628; doi:10.1038/srep38860)
Supplement: Supplementary Information [file srep38860-s1.pdf]

Improved genome-scale multi-target virtual screening via a novel collaborative filtering approach to cold-start problem

Hansaim Lim,[†] Paul Gray,[‡] Lei Xie[†,§] and Aleksandar Poleksic*[‡]*

[†]Department of Computer Science, Hunter College, The City University of New York, New York, New York 10065, United States

[‡]Department of Computer Science, University of Northern Iowa, Cedar Falls, Iowa 50614, United States

[§]Ph.D. Program in Computer Science, Biochemistry and Biology, The Graduate Center, The City University of New York, New York, New York 10065, United States

Supplementary Information

	Description	1st step of the algorithm	2nd step of the algorithm
$\boldsymbol{\lambda_F = \lambda_G}$	regularization parameters for latent matrices	trained	trained
$\boldsymbol{\lambda_M}$	Targets regularization	trained	trained
$\boldsymbol{\lambda_N}$	drugs regularization	trained	trained
$\boldsymbol{r}$	rank	$\min(m, n)$ (capped at 150)	same
$\boldsymbol{w_{i,j}}$	weight on $r_{i,j}$	1 if $r_{i,j} = 0$ and 6 otherwise	same as in the first step if $0.05 \leq p_{i,j} \leq 0.95$; otherwise increased by 1
$\boldsymbol{q_{i,j}}$	impute value for $r_{i,j}$	0	$1 - r_{i,j}$ if $p_{i,j} \geq 0.95$; otherwise $\max(p_{i,j} - r_{i,j}, 0)$
$\boldsymbol{J}$	number of columns/rows to include in weighted profile	5	same
$\boldsymbol{v}$	contribution of the column/row to its own weighted profile	0 for small and 0.1 for large data sets	same

Table S1. Parameters employed in the COSINE algorithm.

	Proteins	*Chemicals*	*Interactions*
N. Recept.	26	54	90
GPCRs	95	223	635
Ion Ch.	204	210	1476
Enzymes	664	445	2926

Table S2. Yam08 data set.

	Proteins	*Chemicals*	*Interactions*
N. Recept.	22	27	44
GPCRs	84	105	314
Ion Chann.	146	99	776
Enzymes	478	212	1515

Table S3. Yam10 data set.

	Chem08	*Pharm10*	*COSINE*
N. Recept.	0.814	0.830	<u>0.884</u>
GPCR	0.811	0.812	<u>0.834</u>
Ion Chann.	0.692	0.731	<u>0.823</u>
Enzyme	0.821	0.845	<u>0.890</u>
AVERAGE	**0.785**	**0.805**	**<u>0.858</u>**

Table S4. AUC scores in 5-fold CV on Yam10 data set. The best results are underlined. Yamanishi 2008 algorithm and its improved 2010 version are abbreviated Chem08 and Pharm10, respectively.

Ligands per Target	*Hidden Interactions*	*Max Chem. Similarity*	*Hidden Interactions*
1-5	304	0.5-0.6	465
6-10	326	0.6-0.7	609
11-15	278	0.7-0.8	538
16-20	290	0.8-0.9	247
>21	2426	0.9-1.0	45

Table S5. ZINC test sets.

	MATRIX SIZE	*NRLMF*	*COSINE*	*NRMLF/entry*	*COSINE/entry*
N. Recept.	1404	0.13s	0.06s	0.09ms	0.04ms
GPCRs	21185	1.72s	0.83s	0.08ms	0.04ms
Ion Chann.	42840	3.06s	3.37s	0.07ms	0.08ms
Enzymes	295480	27.5s	41.11s	0.09ms	0.14ms

Table S6. Running times of NRMLF and COSINE. Columns 2 and 3 represent the running time of NRLMF and COSINE, respectively. The last two columns give the running times per interaction matrix entry.

Parameter settings

COSINE avoids extensive parameter training by globally setting some of the algorithm's parameters, while sacrificing little accuracy. In fact, the only trained parameters are the three regularization parameters. Specifically, we set the rank globally to $r = \min(m, n)$ (capped at 150) and the AdaGrad learning rate to 0.5. The remaining parameters ($\lambda_F = \lambda_G, \lambda_M, \lambda_N$) are chosen from $[10^{-2}, 10^{-1}, 10^{0}] \times [10^{-1}, 10^{0}, 10^{1}] \times [10^{-1}, 10^{0}, 10^{1}, 10^{2}]$. Since the minimization procedure in COSINE is run twice for each choice of parameters, the total number of calls to the iterative AdaGrad procedure in COSINE is 3x3x4x2=72 (the corresponding number in NRLMF is 960).

In contrast to many other algorithms which employ a constant number of iterations, irrespective of the data set under consideration, the number of iterations in COSINE is generally much lower and is a function of the size of the interaction matrix ($[min(100,30 + 0.0016mn)]$). Thus, only about 30 iterations of AdaGrad are used for the Nuclear Receptor data set as opposed to 100 iterations for the Enzyme set. For giant sets, the algorithm's accuracy increases with increasing

number of iterations. We found 600 iterations to be an acceptable tradeoff between the speed and accuracy on the ZINC dataset.
